# Supplementary material for: Update on Prevalence of Pain in Patients with Cancer 2022: A Systematic Literature Review and Meta-Analysis
Source: Cancers (Basel). 2023 Jan 18;15(3):591. doi: 10.3390/cancers15030591 (PMC9913127; doi:10.3390/cancers15030591)
Supplement: Supplementary file 1 [file cancers-15-00591-s001.zip › Supplemental S1.pdf]

Supplemental S1

Search strategy

PubMed

#1 (Neoplasms [Mesh Terms])
#2 (cancer* [Title/Abstract])
#3 (neoplasm* [Title/Abstract])
#4 (neoplastic* [Title/Abstract])
#5 (tumor* [Title/Abstract])
#6 (tumour* [Title/Abstract])
#7 (oncolog* [Title/Abstract])
#8 #1 OR #2 OR #3 OR #4 OR #5 OR #6 OR #7
#9 (Palliative care [MeSH Terms])
#10 (Terminal Care [MeSH Terms])
#11 ("Pain [MeSH Terms])
#12 ("Cancer Pain" [Mesh Terms])
#13 (pain* [Title/Abstract])
#14 (symptom* [Title/Abstract])
#15 (palliat* [Title/Abstract])
#16 (hospice* [Title/Abstract]
#17 ("supportive care" [Title/Abstract])
#18 (terminal* [Title/Abstract])
#19 ("end-of-life" [Title/Abstract])
#20 (dying [Title/Abstract])
#21 (endstage* [Title/Abstract])
#22 (end-stage* [Title/Abstract])
#23 #9 OR #10 OR #11 OR #12 OR #13 OR #14 OR #15 OR #16 OR #17 OR #18 OR #19 OR #20 OR #21 OR #22
#26 (Prevalence [MeSH Terms])
#27 (prevalence* [Title/Abstract])
#28 #26 OR #27
#29 #8 AND #23 AND #26
Filter 2014-2021 AND age 19+ years == 3227

CINAHL

#1 (MH "Neoplasms")
#2 (cancer*])
#3 (neoplasm*)
#4 (neoplastic*)
#5 (tumor*)
#6 (tumour*)
#7 (oncolog*)
#8 #1 OR #2 OR #3 OR #4 OR #5 OR #6 OR #7
#9 (MH "Palliative care")
#10 (MH "Terminal care")
#11 (MH "Pain")
#12 (MH "Cancer pain")
#13 (pain*)
#14 (symptom*)
#15 (palliat*)
#16 (hospice*)
#17 ("supportive care")
#18 (terminal*)

#19 ("end-of-life")
#20 (dying)
#21 (endstage*)
#22 (end-stage*)
#23 #9 OR #10 OR #11 OR #12 OR #13 OR #14 OR #15 OR #16 OR #17 OR #18 OR #19 OR #20 OR #21 OR #22
#24 (MH “Prevalence”)
#25 (prevalence*)
#26 #24 OR #25
#27 #8 AND #23 AND #26
Filter 2014-2021, ≥19 years == 1200

Cochrane

#1 MeSH descriptor: [Neoplasms] this term only
#2 (cancer*)
#3 (neoplasm*)
#4 (neoplastic*)
#5 (tumor*)
#6 (tumour*)
#7 (oncolog*)
#8 #1 OR #2 OR #3 OR #4 OR #5 OR #6 OR #7
#9 MeSH descriptor: [Palliative care] this term only
#10 MeSH descriptor: [Terminal care] this term only
#11 MeSH descriptor: [Pain] this term only
#12 MeSH descriptor: [Cancer pain] this term only
#13 (pain*)
#14 (symptom*)
#15 (palliat*)
#16 (hospice*)
#17 (‘supportive care’)
#18 (terminal*)
#19 ('end-of-life’)
#20 (dying)
#21 (endstage*)
#22 (end-stage*)
#23 #9 OR #10 OR #11 OR #12 OR #13 OR #14 OR #15 OR #16 OR #17 OR #18 OR #19 OR #20 OR #21 OR #22
#24 MeSH descriptor: [Prevalence] this term only
#25 ((prevalence*)): ti,ab,kw
#26 #24 OR #25
#26 #8 AND #23 AND #26
Filter 2014-2021 == 716

Embase
#1 'malignant neoplasm'/exp
#2 cancer*:ab,ti
#3 neoplasm*:ab,ti
#4 neoplastic*:ab,ti
#5 tumor*:ab,ti
#6 tumour*:ab,ti
#7 oncolog*:ab,ti
#8 #1 OR #2 OR #3 OR #4 OR #5 OR #6 OR #7
#9 ‘cancer therapy'/exp
#10 'palliative therapy'/exp
#11 'terminal care'/exp

#12 'pain assessment'/exp
#13 'pain'/exp
#14 pain*:ab,ti
#15 symptom*:ab,ti
#16 palliat*:ab,ti
#17 hospice*:ab,ti
#18 ‘supportive care’:ab,ti
#19 terminal*:ab,ti
#20 'end-of-life':ab,ti
#21 dying:ab,ti
#22 endstage*:ab,ti
#23 'end-stage*':ab,ti
#24 #9 OR #10 OR #11 OR #12 OR #13 OR #14 OR #15 OR #16 OR #17 OR #18 OR #19 OR #20 OR #21 OR #22 OR #23
#25 ‘prevalence’/exp
#26 prevalence*:ab,ti
#27 #25 OR #26
#31 #8 AND #24 AND #27
Filter 2014-2021; Adult:18+ years; AND all publication types except Conference abstracts, Editorials, Notes, Chapters, Article in Press and Letters == 8340
