# Supplementary material for: Update on Prevalence of Pain in Patients with Cancer 2022: A Systematic Literature Review and Meta-Analysis
Source: Cancers (Basel). 2023 Jan 18;15(3):591. doi: 10.3390/cancers15030591 (PMC9913127; doi:10.3390/cancers15030591)
Supplement: Supplementary file 1 [file cancers-15-00591-s001.zip › Supplemental S2.pdf]

Supplemental S2

Table S2.1: Pain prevalence in treatment-naïve cancer patients.

							% Pain					
Study	Quality	Continent[a]	Setting[b]	Cancer[c]	Mean age	Sample	None	Mild	Moderate	ModSev[d]	Severe	Overall
Abbas, 2020 [326]	9	3	5	10	59.6	100						67
Alkhayyat, 2021 [327]	12	1	1,2	6		2020						63.9
Baburao, 2015 [328]	7	3	2	4		96						41.66
Bassey, 2014 [329]	9	5	2	2	30.5	146						15.9
Comelli, 2017 [330]	10	2	2	11		205						14.6
Dai, 2017 [331]	13	3	1	1		262	38.9					61.1
De Carvalho, 2020 [332]	7	4	2	2	32.2	152						23.7
Du Plessis, 2020 [333]	13	5	1	8	56.39	31						22.6
Glover – a, 2019 [334]	11	1	2	6		92260						41.1
Glover – b, 2019 [334]	11		2	6		1680						53.0
Hanna, 2015 [335]	12	1		2		748			7	28	21	
Hlapane, 2021 [336]	12	1	1,2	9	48.2	86						34.9
Hu, 2018 [337]	9	5	2	4		3203						6.9
Iglesias, 2021 [338]	9	3	2	2	72.3	53						45
Khawaja, 2021 [339]	12	3		2	52.08	1067				42.1		67.5
Kkrishnappa, 2016 [340]	9	3	2	6	59.7	29						13.8
Kono, 2019 [341]	9	3		6		72						50
Kuguyo, 2021 [342]	10	5	2	9	52	410						68
Kurnatowski, 2014 [343]	7	2		2	63.1	44						6.98
Lal, 2020 [344]	11	1	1	6		4530						47.0
Marchegiani, 2019 [345]	14	2	2	6		184	64.8[e]			35.2		
Mohme, 2017 [346]	9	2		11	60.7	230						20.9
Paganini Piazzolla – a, 2015 [347]	9	4	2	9		116						47
Paganini Piazzolla – b, 2015 [347]	9	4	2	9		100						68
Pakish, 2016 [348]	14	1	2	9		75						64.0
Philip – a, 2019 [349]	10	1	2	5	65.4	2170						30.2
Philip – b, 2019 [349]	10	1	2	6	66.8	379						28.1
Philip – c, 2019 [349]	10	1	2	7	67.8	1610						16.1
Poursadegh, 2015 [350]	12	3	2	11	51.1	69						18.8
Rudd, 2017 [351]	14	5	2	9	42	300						3.3

Shen, 2018 [352]	13	1	2	11		9319						22.19

[a]1 = North America; 2 = Europe; 3 = Asia; 4 = South America; 5 = Africa; 6 = Australia / New Zealand; [b]1 = inpatient; 2 = outpatient; 3 = patient in a palliative care setting; 4 = all; 5 = other; [c]1 = >3 types of cancer; 2 = head and neck; 3 = esophagus, 4 = bronchus / lung; 5 = breast; 6 = gastro-intestinal; 7 = prostate; 8 = other, urological; 9 = gynaecological; 10 = haematological; 11 = other; [d]moderate–severe pain; [e]none–mild pain.

Table S2.2: Pain prevalence in patients with curative treatment.

							% Pain					
Study	Quality	Continent[a]	Setting[b]	Cancer[c]	Mean age	Sample	None	Mild	Moderate	ModSev[d]	Severe	Overall
Antony, 2016 [353]	14	3	1	1		120						3.7
Berger, 2020 [354]	14	1	2	5	52	204		84.8				84.8
Bianchini, 2016 [355]	14	2	1	2	62.84	164						28.1
Dervis, 2016 [356]	13	3	1	10	49.03	66						12.1
Fatiregun, 2019 [357]	14	5	2	1	47.4	205						49.3
Frowen, 2020 [358]	13	6	1,2	1	59	239	80.3	11.3	3.8	8.4	4.6	20
Germano, 2015 [359]	14	2	2	2		20						90
Kartin – a, 2014 [360]	14	2	2	2		20	0	15	80	85	5	100
Kartin – b, 2014 [360]	14	2	2	2		30	0	3.3	40	96.7	56.7	100
Kurnatowski, 2014 [343]	7	2		2	63.1	44						58.1
Rahnama, 2015 [361]	13	2	2		63.87	58	72.41[e]		27.59	29.31	1.72	
Sahora, 2016 [362]	10	2	2	6		50						50
Takayama, 2021 [363]	14	3	1	6	68.9	246						68
Wagland, 2016 [364]	12	2	2	1		363	54	6	12	40	28	46
Wang, 2021 [365]	14	3	2	2	50.9	169						93.5
Yanque, 2018 [366]	9	4		11	45.36	11						54

[a]1 = North America; 2 = Europe; 3 = Asia; 4 = South America; 5 = Africa; 6 = Australia / New Zealand; [b]1 = inpatient; 2 = outpatient; 3 = patient in a palliative care setting; 4 = all; 5 = other; [c]1 = >3 types of cancer; 2 = head and neck; 3 = esophagus, 4 = bronchus / lung; 5 = breast; 6 = gastro-intestinal; 7 = prostate; 8 = other, urological; 9 = gynaecological; 10 = hematological; 11 = other; [d]moderate–severe pain; [e]none–mild pain.

Table S2.3: Pain prevalence in patients with palliative treatment.

							% Pain					
Study	Quality	Continent[a]	Setting[b]	Cancer[c]	Mean age	Sample	None	Mild	Moderate	ModSev[d]	Severe	Overall
Ahmadi, 2016 [367]	13	2	1,2,4	4	72.9	10822						73.0
Alsirafy, 2016 [368]	13	5		1		89	38	8	21	54	33	62
Bischoff, 2018 [369]	12	1	4			7317				41		40.8
Cuyun Carter, 2017 [370]	11	3	1,2	6	60	122						61
Eriksson, 2016 [371]	13	2	4		83.5	1626	19.7					78.0

Study	Quality	Continent[a]	Setting[b]	Cancer[c]	Mean age	Sample	None	Mild	Moderate	ModSev[d]	Severe	Overall
Family – a, 2018 [372]	9	1		4,5,10	66.3	402						34.6
Family – b, 2018 [372]	9	1		4,5,10	62	98						22.4
Hasegawa, 2016 [373]	14	3	1	1	66.6	45		20.9		27.9		48.8
Iglesias, 2021 [338]	9	2	2	2	72.3	53						29
Langlais, 2019 [374]	13	1	2	10	58.5	1416						52.6
Nnonyelum, 2015 [375]	8	5		10	58.8	135						74
Pimentel, 2015 [376]	14	1	1,2	1		375						24.0
Richter, 2021 [377]	14	1	2	10		239						36
Sandgren, 2018 [378]	14	2	1	1	73	589						42.3
Scharf, 2018 [379]	13	2	2	11		85						42.4
Spence, 2020 [380]	12	1	2	1		607					33.9	
Unseld, 2020 [381]	13	2	2	6		69						11.6
Wangnamthip, 2016 [382]	13	2	2	6		50						14
Yanamandra, 2018 [383]	12	3	1,2	10	36.03	40						55.17
Yi – a, 2018 [384]	13	3	2	4	66.8	337						37.4
Yi – b, 2018 [384]	13	3	2	4	66.8	337						23.7

[a]1 = North America; 2 = Europe; 3 = Asia; 4 = South America; 5 = Africa; 6 = Australia / New Zealand; [b]1 = inpatient; 2 = outpatient; 3 = patient in a palliative care setting; 4 = all; 5 = other; [c]1 = >3 types of cancer; 2 = head and neck; 3 = esophagus, 4 = bronchus / lung; 5 = breast; 6 = gastro-intestinal; 7 = prostate; 8 = other, urological; 9 = gynaecological; 10 = haematological; 11 = other; [d]moderate–severe pain.

Table S2.4: Pain prevalence in patients with either curative or palliative treatment, or treatment intent not specified.

							% Pain					
Study	Quality	Continent[a]	Setting[b]	Cancer[c]	Mean age	Sample	None	Mild	Moderate	ModSev[d]	Severe	Overall
Abid, 2017 [385]	14	1	2	1	57	1333						72.8
Agyemang-Yeboah, 2017 [386]	9	5		6	54	221						38.91
Allgar, 2018 [387]	10	6	2			39	26	33	23	41	18	74
Alsughayer, 2021 [388]	14	3	2	5,6,10	51.8	280						20
Bell, 2020 [389]	13	1	1,2	9	59.5	146						29.5
Bernardes, 2019 [106]e	14	6	1,2	1		125	59	14		28		42
Crossnohere, 2019 [390]	14	1	2	10	54.7	901						33
Hirpara, 2020 [391]	12	1	2	4	69.05	11075					25	
Jazieh, 2018 [392]	9	3		4		259						19.7
Kawazoe, 2020 [393]	9	3	1	1		141						34.8
Kebebew, 2021 [394]	14	5	2	9		385						53.8
Kokkonen, 2019 [395]	13	2	5	1	62.2	297	53	42	5	5	0	47

Kolb, 2018 [396]	13	2	5	1	62.2	281	34	50	11	16	5	66
Ladaninejad, 2019 [397]	13	3	1,2	1	67.82	200	52					48
Lim, 2018 [398]	10	6	1	1	57.5				32.7	40.4	7.7	
Molassiotis, 2019 [399]	14	3	2	1								26.7
Motah, 2021 [400]	9	5	1,2	11		150						88
Neves Duarte Lisboa, 2021 [401]	13	4	1	1	55.6	240						57.9
Parás-Bravo, 2017 [402]	12	2	1	1	61.17	402	81.6					18.4
Porta-Sales – a, 2015 [403]	14	2	2	1		437						41.6
Porta-Sales – b, 2015 [403]	14	2	1	1		627						61.7
Pozzar – a, 2021 [404]	14	1	2	1	64.1	43						11.6
Pozzar – b, 2021 [404]	14	1	2	1	61.2	101						22.8
Pozzar – c, 2021 [404]	14	1	2	1	55.9	68						30.9
Pozzar – d, 2021 [404]	14	1	2	1	54.6	20						50.0
Santos, 2021 [405]	14	2	1,2	6	69	41						51.2
Williams, 2016 [406]	14	1		1		550	14					86

[a]1 = North America; 2 = Europe; 3 = Asia; 4 = South America; 5 = Africa; 6 = Australia / New Zealand; [b]1 = inpatient; 2 = outpatient; 3 = patient in a palliative care setting; 4 = all; 5 = other; [c]1 = >3 types of cancer; 2 = head and neck; 3 = esophagus, 4 = bronchus / lung; 5 = breast; 6 = gastro-intestinal; 7 = prostate; 8 = other, urological; 9 = gynaecological; 10 = haematological; 11 = other; [d]moderate–severe pain; [e]study including two samples, scoring respectively <15 points and ≥15 points.

Table S2.5: Pain prevalence in patients after curative treatment.

							% Pain					
Study	Quality	Continent[a]	Setting[b]	Cancer[c]	Mean age	Sample	None	Mild	Moderate	ModSev[d]	Severe	Overall
Berger, 2020 [354]	14	1	2	5		173		60.1				60.1
Beyaz, 2016 [407]	14	2	2	5	55.2	131	35.9	50.4	13.7	13.7	0	64.1
Capelan, 2017 [155]e	9	2	2	5		411						14
Cardoso, 2015 [408]	14	4	2	2		167	42.5	31.7		25.7		57.4
Cox-Martin, 2020 [409]	14	1	2	1		1702						17
Fuchs – a, 2016 [410]	12	2	2	3	67	71						87.1
Fuchs – b, 2016 [410]	12	2	2	3	73	52						78
Huang, 2017 [411]	14	1	2	1		604						73.47
Jefford – a, 2017 [412]	12	6	2	5		1006						52
Jefford – b, 2017 [412]	12	6	2	6		992						34
Jefford – c, 2017 [412]	12	6	2	11		1024						27
Jefford – d, 2017 [412]	12	6	2	10		586						35
Jefford – e, 2017 [412]	12	6	2	7		1066						22

Jiang, 2019 [413]	14	1	2	1		4526						34.6
Kimberg, 2015 [414]	14	1	2	1	31.6	1304						25
Kurnatowski, 2014 [343]	7	2		2	63.1	44						12
Lang, 2021 [415]	13	1	2	5		63						49.2
Maass, 2020 [416]	14	2	2	5		350						9.1
Maguire, 2019 [417]	14	2	2	7	71.57	3384	59					37
Mahmood, 2021 [418]	14	3	2	5	58.54	35						41
McDonough, 2021 [419]	12	1	2	5		195						55.0
Mehdizadeh, 2020 [420]	10	2	2	2	70							79.1
Moreno, 2019 [421]	14	1		6	55.03	70				19.7		
Mustafa Ali, 2017 [422]	11	1	2	5		1126						24
Oancea, 2014 [423]	13	1	2	1		1863	66.3	16.1	9.4	16.0	6.6	32.1
Olsson, 2020 [424]	11	1	2	1	34	911						21.7
Pezdirec, 2019 [425]	14	2	2	2	65.4	109						27.5
Romero, 2016 [426]	8	2	2	5		1057						11.3
Salani, 2014 [427]	14	1	2	9	62.9	305						27
Soares, 2014 [428]	13	4		5	53	200						27.5
Wulff-Burchfield, 2019 [429]	14	1	2	2	61.9	92						53.3
Yen, 2020 [430]	13	1	2	10		1218						73.2

[a]1 = North America; 2 = Europe; 3 = Asia; 4 = South America; 5 = Africa; 6 = Australia / New Zealand; [b]1 = inpatient; 2 = outpatient; 3 = patient in a palliative care setting; 4 = all; 5 = other; [c]1 = >3 types of cancer; 2 = head and neck; 3 = esophagus, 4 = bronchus / lung; 5 = breast; 6 = gastro-intestinal; 7 = prostate; 8 = other, urological; 9 = gynaecological; 10 = haematological; 11 = other; [d]moderate–severe pain; [e]study including two samples, scoring respectively <15 points and ≥15 points.

Table S2.6: Pain prevalence in patients without feasible anti-cancer treatment.

							% Pain					
Study	Quality	Continent[a]	Setting[b]	Cancer[c]	Mean age	Sample	None	Mild	Moderate	ModSev[d]	Severe	Overall
Bandeali, 2020 [431]	7	1	4	1	67	54						78
Covarrubias-Gómez, 2014 [432]	12	4	2	1	60.7	38						71
Díez-Manglano, 2020 [433]	7	2,4	3			194						55.2
Effendy – a, 2015 [434]	13	2	2	1	58	94						67
Effendy – b, 2015 [434]	13	3	2	1	49.3	180						71.1
Goto, 2017 [435]	9	3		11	65.2	224						56.6
Jaime-Pérez, 2020 [436]	9	4	3	10		81						69.7
Jakobsen, 2020 [437]	14	2	4	1	73.7	440						30.5
Janberidze, 2016 [438]	9	2	4			1363	20.1				33.4	79.4

Mercadante, 2015 [439]	13	2	3	1	73.5	412	40.9	46.7	8.3	12.4	4.1	59.1
Parra Palacio, 2018 [440]	14	4	3	1	61.1	66						50
Prado, 2018 [441]	9	4	4	1	67.8	203						11
Romem, 2015 [442]	13	1	3		73.7	47		8.5		83		91.5
Seiler – a, 2020 [443]	11	2	3	1	66.4	229						90
Seiler – b, 2020 [443]	11	2	3	1	64.5	181						83
Soares, 2018 [444]	14	4	3	1	73	54		11		46		57

[a]1 = North America; 2 = Europe; 3 = Asia; 4 = South America; 5 = Africa; 6 = Australia / New Zealand; [b]1 = inpatient; 2 = outpatient; 3 = patient in a palliative care setting; 4 = all; 5 = other; [c]1 = >3 types of cancer; 2 = head and neck; 3 = esophagus, 4 = bronchus / lung; 5 = breast; 6 = gastro-intestinal; 7 = prostate; 8 = other, urological; 9 = gynaecological; 10 = haematological; 11 = other; [d]moderate–severe pain.

Table S2.7: Pain prevalence including patients in different phases of treatment.

							% Pain					
Study	Quality	Continent[a]	Setting[b]	Cancer[c]	Mean age	Sample	None	Mild	Moderate	ModSev[d]	Severe	Overall
Aboumrad, 2018 [445]	14	1	5	1		64						41.0
Aghdassi, 2018 [446]	11	2	5	6	66.86	104						38.5
Aldossary, 2018 [447]	13	3	1,2	6	62.4	76						84.0
Aljumah, 2016 [448]	9	3		6	65	235						28.1
Alsalamah, 2020 [449]	14	3		2	51.4	110						15.5
Alzghoul, 2021 [450]	13	1	5	10		43						37.2
Anvari, 2014 [451]	9	3	1	11	47.8	23						43.5
Bentley, 2019 [452]	11	2	2	1	65	236						17
Bubis, 2021 [453]	13	1	2	6		788				32		
Bunduc – a, 2018 [454]	9	2		6		24	40					60
Bunduc – b,2018 [454]	9	2		6		124	77.1					22.9
de Melo, 2019 [455]	14	4		2		130						36.2
de Sire, 2020 [456]	12	2	2	5	60	52						67.3
Diallo, 2021 [457]	13	5	1	11	47.4	229						91.7
Dubé – a, 2018 [458]	14	1	6	1		210732						67.9
Dubé – b, 2018 [458]	14	1	6	1		81911						55.9
Dubé – c, 2018 [458]	14	1	6	1		44090						41.8
Guérin, 2015 [459]	11	1	2	2,4	54.8	213						23.9
Hallet, 2019 [460]	13	1	2	11		2721				19		
Hamieh, 2018 [461]	14	3	5	1	54.7	400						29.8
Haynes-Lewis, 2018 [462]	14	1	2	1	70.7	100						46

Iadeluca, 2017 [463]	13	1	2	1		1910						33.7
Jeon, 2021 [464]	14	6	2	2	51.1	81						11.1
Kamieniarz, 2020 [465]	7	2	2	11	59	27						3.7
Miyashita, 2015 [466]	14	3	4	1	60.7	312	37.8	44.6	10.6	15.4	4.8	59.9
O'Conner, 2014 [467]	11	4	2	11	53.2	461						32
Parrozzani, 2020 [468]	13	2	1,2	11		15						80
Phanphaisarn, 2016 [469]	9	3	2	1	57.6	2263						27.1
Pimentel, 2015 [470]	11	1	1	1	80	8094		25.2	36.1	49.6	13.5	74.8
Presley, 2020 [471]	14	4	5	1	75	9105			46	68.8	22.6	
Roila, 2019 [472]	11	2	1,2	1	62.21	1394	78.2					21.8
Sadik, 2014 [473]	13	3	2	1	57.9	408						17.8
Saito, 2019 [474]	10	3	2	4	66.5	124						57.1
Santos, 2016 [475]	9	4	2	2		76						69.7
Somboon, 2014 [476]	9	3	5	11	57.4	125						44.8
Song, 2017 [477]	13	1		11		358						35
Taghizadeh, 2018 [478]	14	3		1	54	256						59.4
Teo, 2018 [479]	14	3	1,2	9	49.86	104						25
van Londen, 2014 [480]	14	1	2	5	53.1	1013						33
Wei-Yun, 2016 [481]	12	3	1	1	59.3	24430			22.3	41.6	18.3	
Yassin, 2020 [482]	11	3	2	10	57.7	843						43.1
Yates – a, 2015 [483]	13	6	2	1	50.3	263						73
Yates – b, 2015 [483]	13	6	2	1	70.1	330						54.9
Yenugadhati, 2018 [484]	14	3	2	1	51.8	375						44
Yung, 2019 [485]	13	3	2	11	58	605						5.3

[a]1 = North America; 2 = Europe; 3 = Asia; 4 = South America; 5 = Africa; 6 = Australia / New Zealand; [b]1 = inpatient; 2 = outpatient; 3 = patient in a palliative care setting; 4 = all; 5 = other; [c]1 = >3 types of cancer; 2 = head and neck; 3 = esophagus, 4 = bronchus / lung; 5 = breast; 6 = gastro-intestinal; 7 = prostate; 8 = other, urological; 9 = gynaecological; 10 = haematological; 11 = other; [d]moderate–severe pain.
