# Supplementary material for: Update on Prevalence of Pain in Patients with Cancer 2022: A Systematic Literature Review and Meta-Analysis
Source: Cancers (Basel). 2023 Jan 18;15(3):591. doi: 10.3390/cancers15030591 (PMC9913127; doi:10.3390/cancers15030591)
Supplement: Supplementary file 1 [file cancers-15-00591-s001.zip › Supplemental S3.pdf]

Supplemental S3

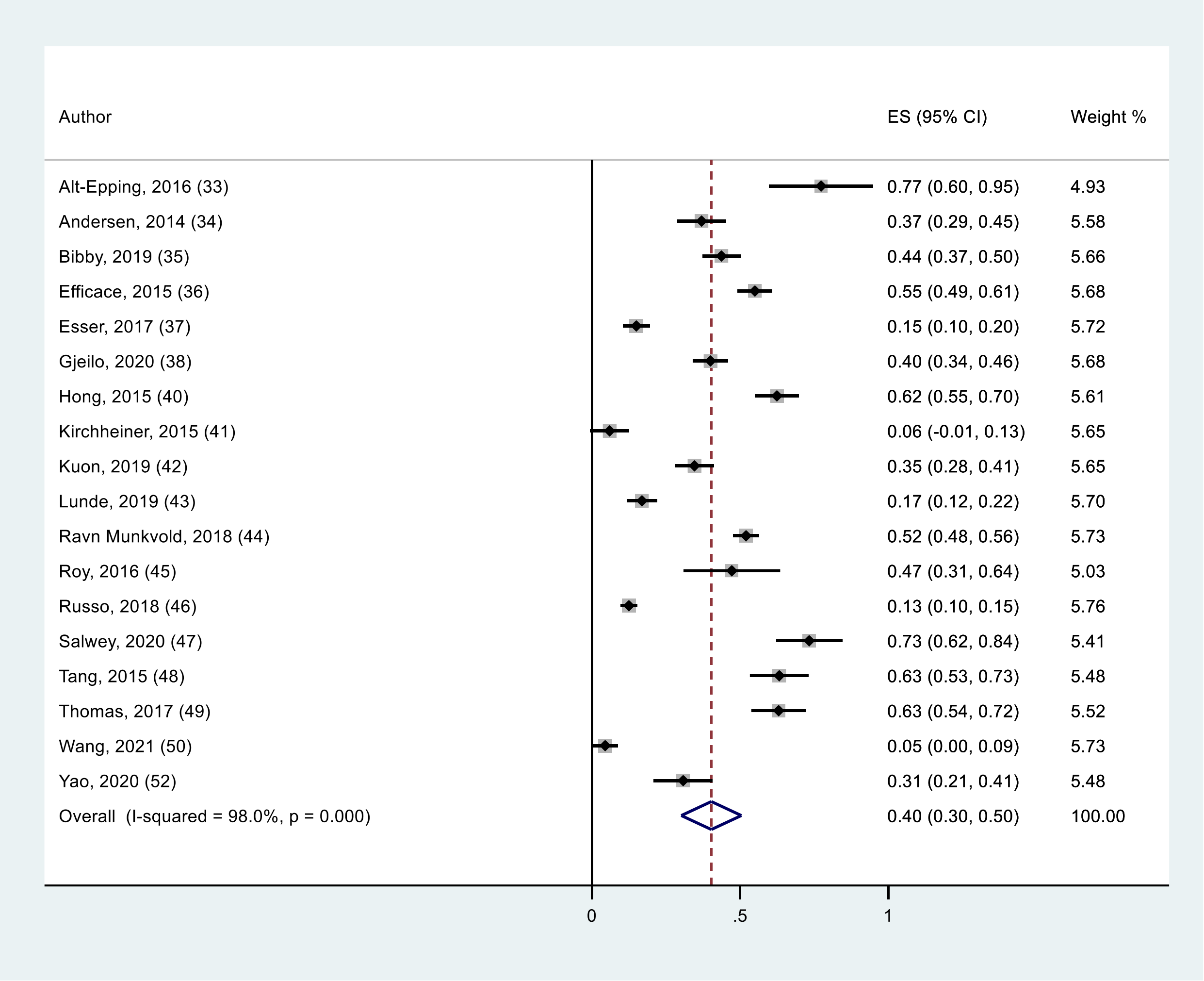

Figure S3.1: Forest plot of pain prevalence in treatment-naïve cancer patients (Group 1) [33–50,52].

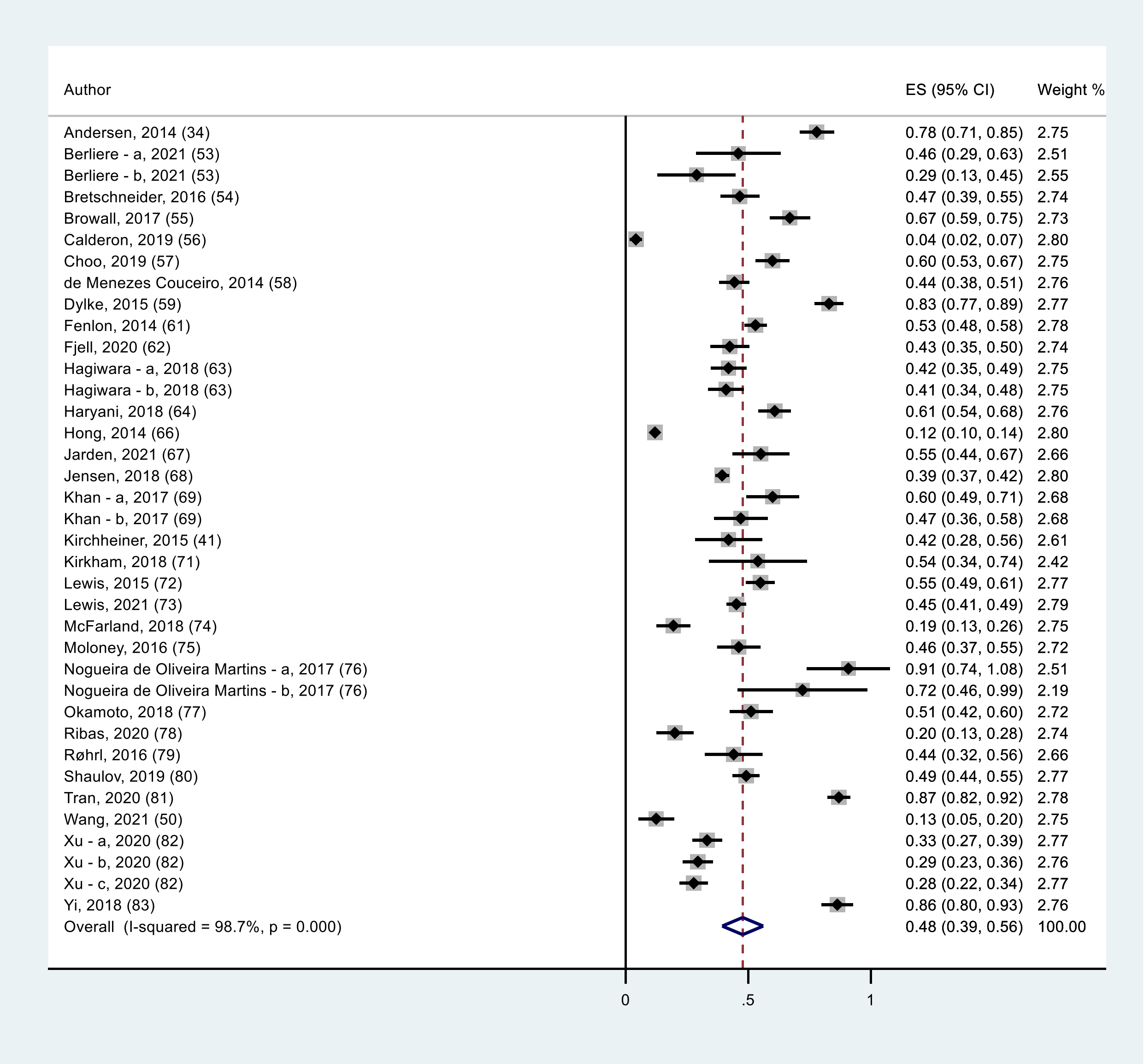

Figure S3.2: Forest plot of pain prevalence in patients with curative treatment (Group 2).

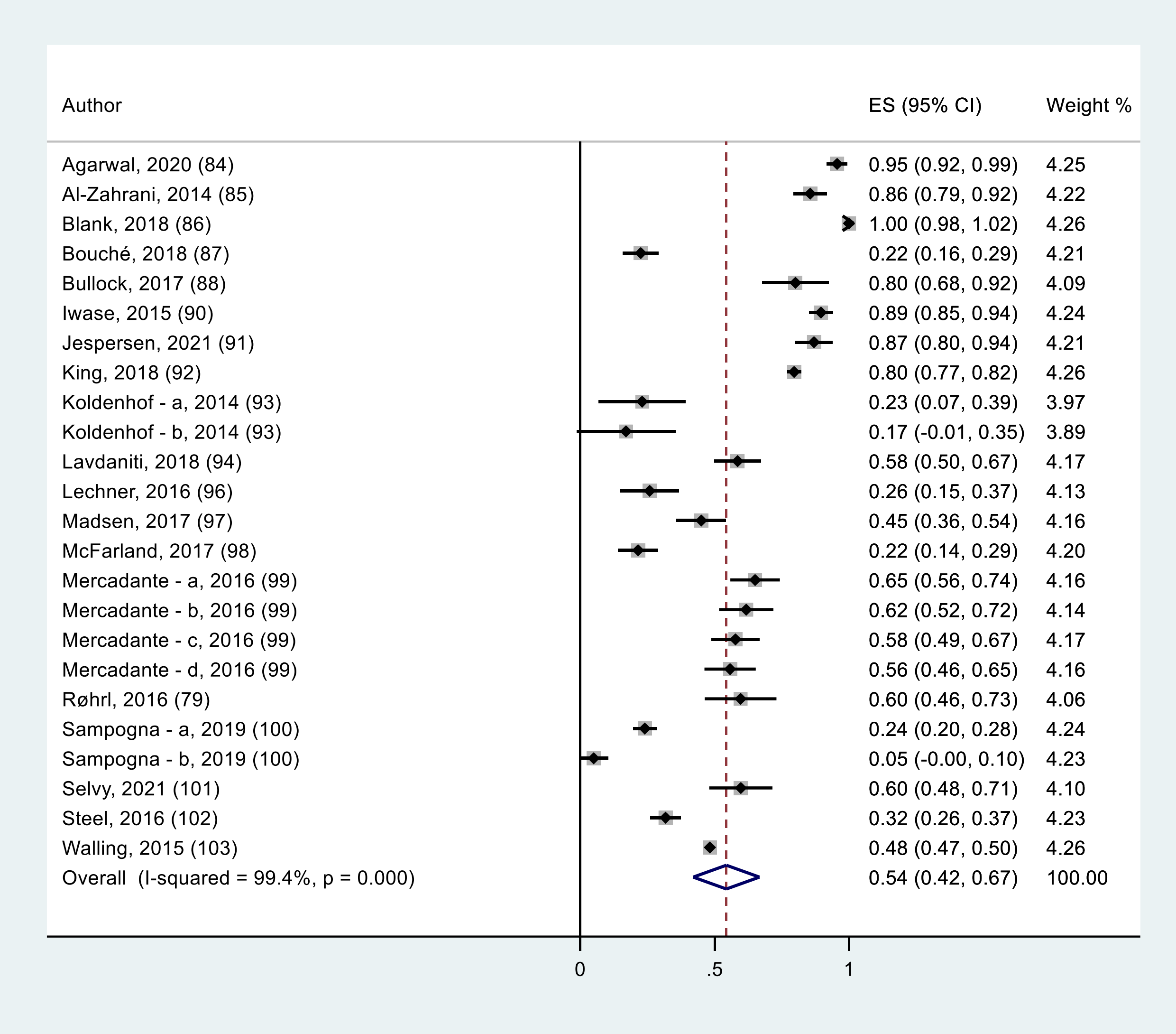

Figure S3.3: Forest plot of pain prevalence in patients with palliative treatment (Group 3).

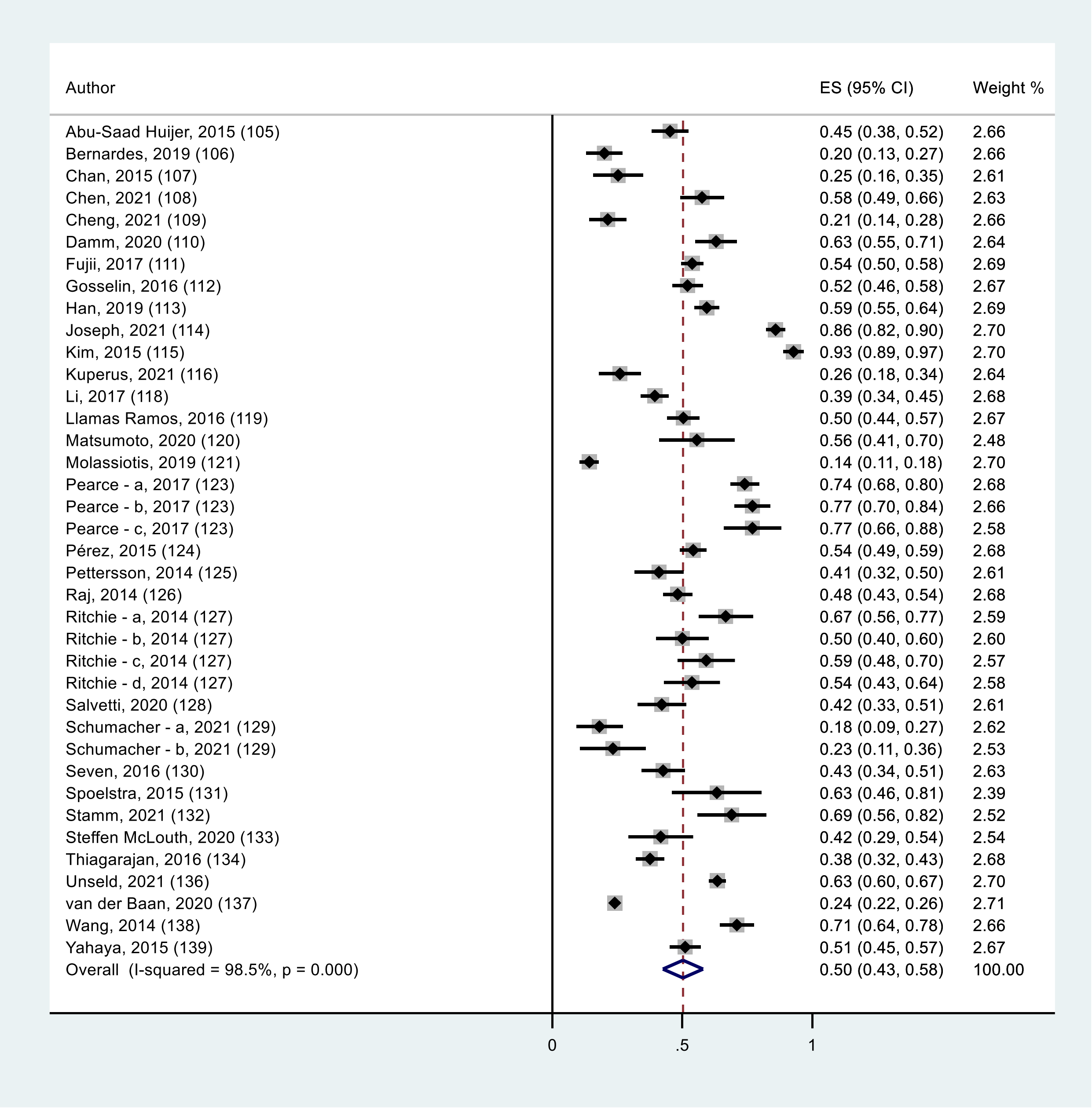

Figure S3.4: Forest plot of pain prevalence in patients with either curative or palliative treatment, or treatment intent not specified (Group 4).

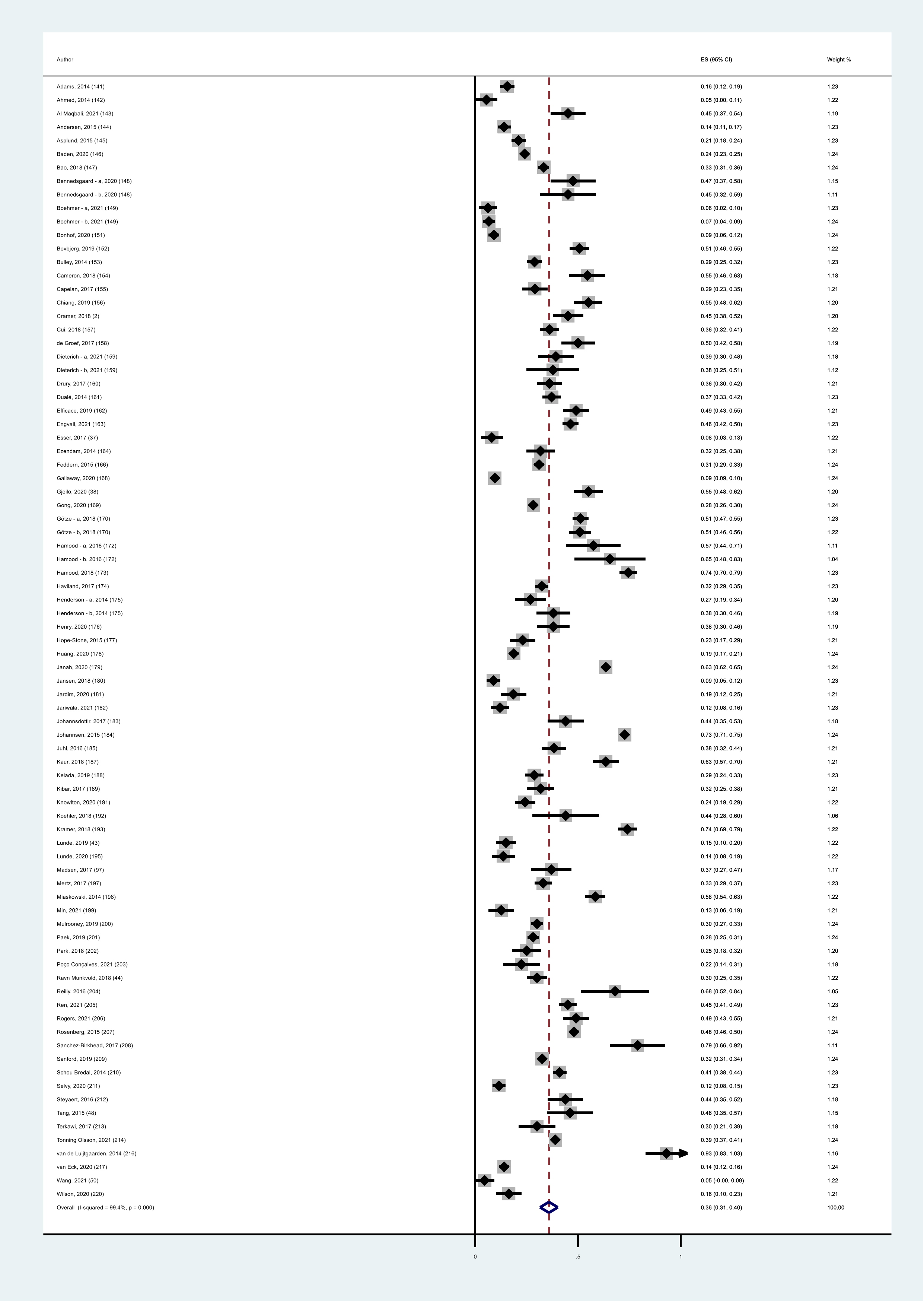

Figure S3.5: Forest plot of pain prevalence in patients after curative treatment (Group 5).

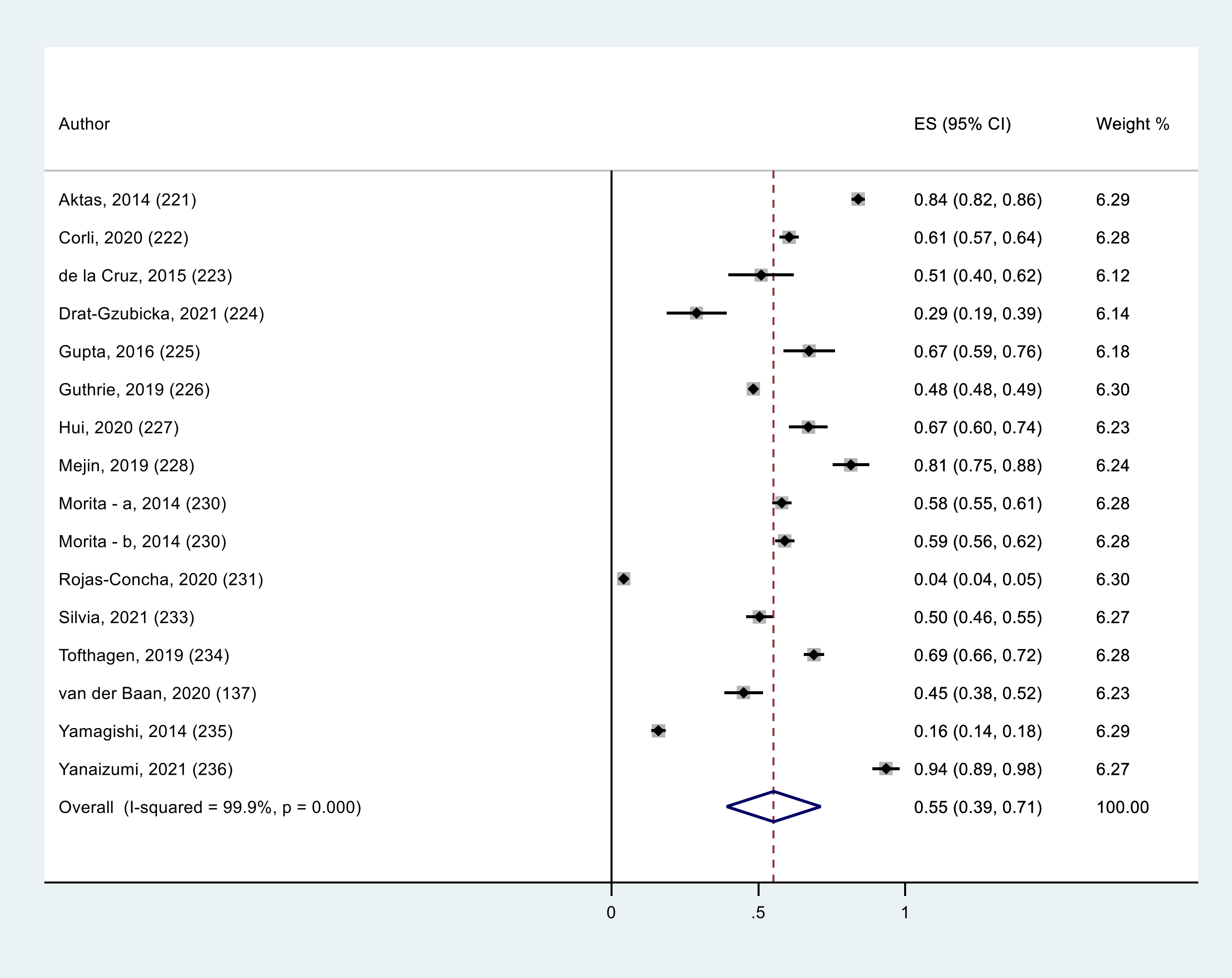

Figure S3.6: Forest plot of pain prevalence in patients without feasible anti-cancer treatment (Group 6).

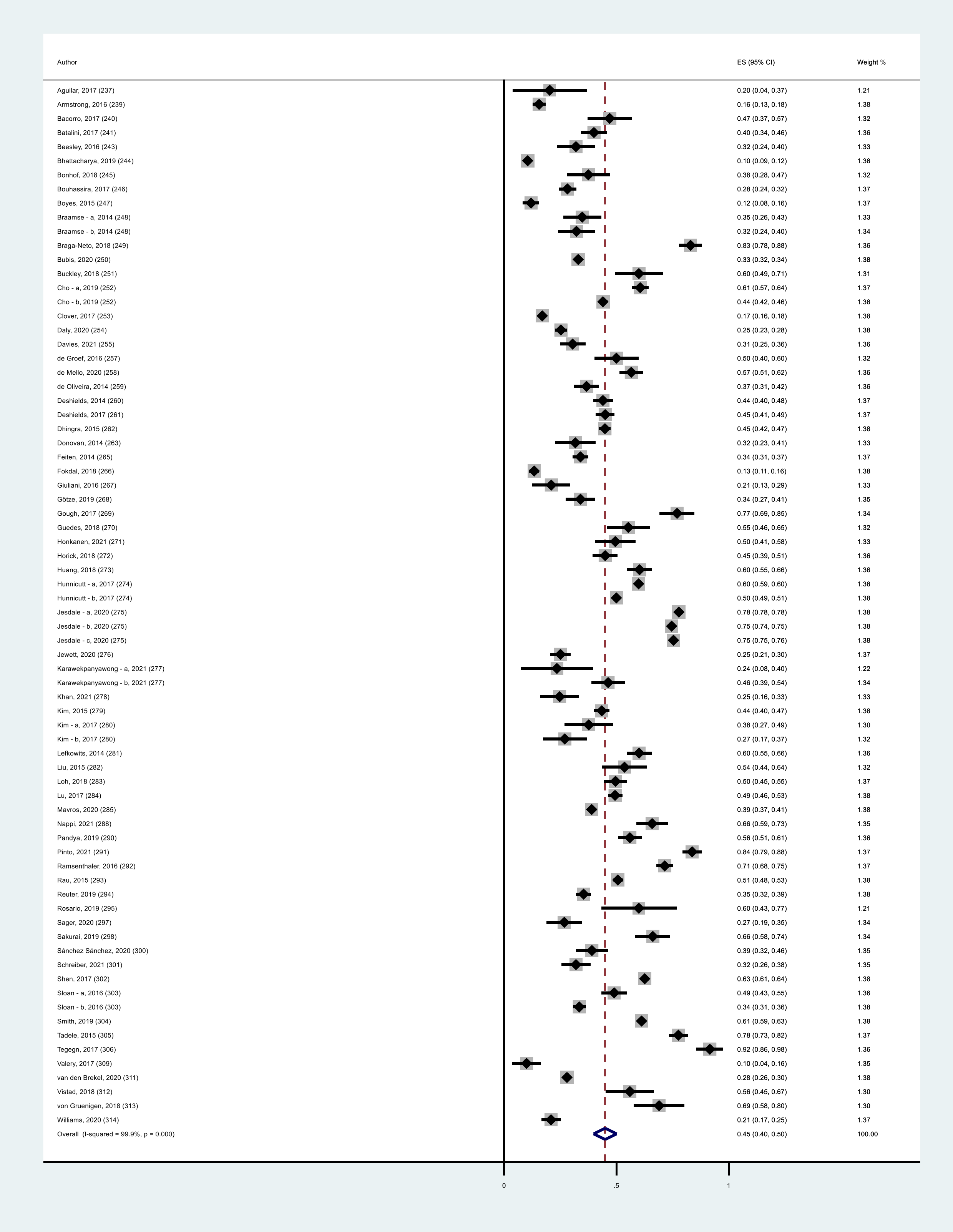

Figure S3.7: Forest plot of pain prevalence including patients in different phases of treatment (Group 7).
